# Different Prognostic Values of Plasma Epstein-Barr Virus DNA and Maximal Standardized Uptake Value of ^18^F-FDG PET/CT for Nasopharyngeal Carcinoma Patients with Recurrence

**DOI:** 10.1371/journal.pone.0122756

**Published:** 2015-04-08

**Authors:** Ting Shen, Lin-Quan Tang, Dong-Hua Luo, Qiu-Yan Chen, Pei-Jing Li, Dong-Mei Mai, Shan-Shan Guo, Li-Ting Liu, Chao-Nan Qian, Xiang Guo, Mu-Sheng Zeng, Hao-Yuan Mo, Hai-Qiang Mai

**Affiliations:** 1 The State Key Laboratory of Oncology in South China, Collaborative Innovation Center for Cancer Medicine, Sun Yat-sen University Cancer Center, Guangzhou, P.R. China; 2 Department of Nasopharyngeal Carcinoma, Sun Yat-sen University Cancer Center, Guangzhou, P.R. China; The University of North Carolina at Chapel Hill, UNITED STATES

## Abstract

**Purpose:**

To evaluate and compare the prognostic value of Epstein-Barr virus (EBV) DNA and maximal standard uptake values (SUV_max _) of ^18^F-fluoro-2-deoxy-D-glucose positron emission tomography (^18^F-FDG-PET) in subgroups of nasopharyngeal carcinoma (NPC) patients with locoregional or distant recurrence.

**Patients and Methods:**

A total of 194 patients with recurrent NPC (locoregional recurrence: 107, distant recurrence: 87) were enrolled. Patients took evidence of recurrence performed with ^18^F-FDG-PET and an EBV DNA test before salvage treatment. Clinical parameters, the status of EBV DNA and the value of SUV_max_ were used for survival analysis using the Kaplan-Meier method and the Cox proportional hazards regression model.

**Results:**

In the subgroup of patients with locoregional recurrence, patients with SUV_max_<8.65 had significantly better overall survival (OS) (P=0.005) compared with the patients with SUV_max_ ≥8.65. However, both elevated EBV DNA load (≥21,100 copies/ml) and distant SUV_max_ (≥13.55) were significantly associated with worse OS compared with the patients with EBV DNA <21,100 copies/ml or distant SUV_max_ <13.55 for the subgroup with distant recurrence (P=0.015 and P=0.006, respectively). The predictive ability of EBV DNA was superior to that of SUV_max_ (P=0.062). Multivariate analysis showed that SUV_max_ was only an independent prognostic factor for OS in patients with locoregional recurrence (P=0.042), whereas EBV DNA independently predicted OS for the patients with distant recurrence (P=0.007). For those patients with undetectable EBV DNA, SUV_max_<8.65 was still an independent favorable prognostic factor (P=0.038).

**Conclusions:**

SUV_max_ is a useful biomarker for predicting OS in nasopharyngeal carcinoma patients with locoregional recurrence or with undetectable EBV DNA. Both distant SUV_max_ and EBV DNA appear to be independent predictors of OS in patients with distant recurrence; however, the predictive ability of EBV DNA was superior to that of SUV_max_.

## Introduction

Nasopharyngeal carcinoma (NPC) is common in southern China, especially in the Guangdong province, where rates range from 20 to 30 cases per 100,000 population.[1, 2] Radiotherapy is the primary treatment modality for NPC, and concurrent chemo-radiotherapy (CCRT) with or without adjuvant chemotherapy is the standard treatment regimen for locoregional advanced disease.[3, 4] Despite significant improvements in local control due to advances in radiotherapy and combined modality treatments, local recurrence and distant metastasis remain the predominant mode of failure in patients with advanced NPC.[5, 6] Locoregional recurrent NPC re-irradiated by IMRT can still have an encouraging outcome; the 5-year local control rate (LCR) and overall survival rate (OS) for recurrent stage I, II, III and IV were 80.0%, 85.0%, 80.0% and 78.7% and 71.4%, 62.9%, 35.5% and 30.2%, respectively.[7] However, when distant metastases are present, the median survival for metastatic NPC is only 12–20 months.[8] There remains a mysterious heterogeneity of outcomes for patients with locoregional or distant recurrence. Therefore, the identification of effective prognostic factors that more accurately correlate with treatment outcome would be of great importance for determining which NPC patients might benefit from intense treatment.[9]

Currently, the most important and widely recognized prognostic factor identified to date is the AJCC tumor-node metastasis (TNM) staging system.[10–12] However, previous studies demonstrated that the clinical TNM staging for patients with recurrence and distant metastasis does not always provide a satisfactory prediction.[13] Accordingly, an increasing amount of effort is being devoted to identifying better prognostic markers to supplement the staging system. Epstein-Barr virus DNA is a marker that has long been intensively studied and is considered a useful tool to supplement the TNM system for prognostication in NPC.[14] The prognostication value of EBV DNA in recurrent NPC has been demonstrated in several studies.[15–17] However, in some cases of recurrent NPC, patients presented with undetectable levels of EBV DNA.[18] In addition, although EBV DNA levels were detectable, the amount of data on the EBV DNA load of patients with locoregional recurrent NPC is relatively low.[17]

Recent studies have addressed the role of ^18^F-fluoro-2-deoxy-D-glucose positron emission tomography (^18^F-FDG-PET) maximal standard uptake value (SUV_max_) as a risk stratification marker to predict therapeutic response or outcome in patients with head and neck cancers,[19, 20] lung cancer,[13] and NPC.[9]^,^[21]^,^[22] Notably, studies on the prognostic value of SUV_max_ are mostly focused on the pretreatment SUV_max_ of patients; however, data to evaluate the prognostic role of SUV_max_ for patients with locoregional recurrence or distant recurrence are rare. Furthermore, the comparison of the role of EBV DNA load and SUV_max_ in predicting the outcome of patients with recurrent NPC has not yet been well defined. Therefore, in the present study, we aimed to determine and compare the prediction value of ^18^F-FDG PET/CT scan SUV_max_ and EBV DNA load in locoregional or distant recurrent NPC patients.

## Materials and Methods

### Patients

We retrospectively analyzed initial recurrent NPC patients who were referred to Sun Yat-sen University Cancer Center, Guangzhou, China between July 2007 and December 2013. All patients had received radiotherapy alone, concurrent chemo-radiotherapy (CCRT) or induction chemotherapy followed by CCRT, depending on the stage of disease on initial presentation. When patients clinically indicated with locoregional recurrence or distant recurrence, whole-body ^18^F-FDG PET examination and EBV DNA measurement were performed on each patient before salvage treatment. In addition to ^18^F-FDG PET, magnetic resonance imaging (MRI) of the head and neck and conventional workups, including physical examinations, endoscopy, chest radiography, whole-body bone scans, and abdominal ultrasonography, were performed depending on the status of each patient. These examinations were performed within 2 weeks of enrollment in the study. All enrolled patients received salvage treatment using platinum-based adjuvant chemotherapy. Patients were excluded who met the following criteria: (1) insulin-dependent diabetes or serum glucose levels > 200 mg/dl immediately before the ^18^F-FDG was injected; (2) a history of previous or synchronous malignant tumors; (3) persistent residual NPC; (4) lost during follow-up; (5) incomplete medical records. Finally, a total of 194 patients were enrolled in our study. Eligible patients were divided into two subgroups, one with locoregional recurrence only and one with distant recurrence. The hospital records for each patient were reviewed for demographic and clinical data, including age, gender, family history of tumor, number of metastases, clinical tumor restage, the concentration of plasma EBV DNA and SUV_max_ of ^18^F-FDG PET/CT. The tumor of each patient was restaged according to the seventh American Joint Committee on Cancer (AJCC) TNM staging manual.[23] This study was approved by the independent Institute Research Ethics Committee at the Sun Yat-sen University Cancer Center (SYSUCC, Guangzhou, P. R. China), and written consents were obtained from all participants.

### PET/CT imaging

PET/CT imaging was performed with a combination PET/CT scanner (Discovery ST 16; GE Healthcare, Little Chalfont, United Kingdom) according to published guidelines for tumor imaging with PET/CT.[24] Helical CT was performed from the head to the proximal thigh before PET acquisition according to a standardized protocol. PET/CT scans from the head to the proximal thigh were begun 45 to 60 minutes after the injection of 5.55 MBq/kg of FDG. The PET images were reconstructed with the use of CT data for attenuation correction with an ordered-subset expectation maximization iterative reconstruction algorithm. The standardized FDG uptake value was calculated using the concentration of FDG in the region of interest, as measured by PET, divided by the injected FDG dose and multiplied by body weight as a normalization factor. The SUV_max_ for each patient was used to minimize partial-volume effects. The SUV_max_ values of locoregional and distant metastases were used as SUV_max_ for the locoregional recurrent group and the distant recurrent group, respectively. ^18^F-FDG PET/CT scans were performed on all patients before salvage treatment.

### Plasma EBV DNA measurement

As previously described,[15, 25, 26] patient plasma EBV DNA (EBV DNA) concentrations were routinely measured by q-PCR before salvage treatment.

### Statistical analysis

The Statistical Package for the Social Sciences software (SPSS v. 17, SPSS, Inc., Chicago, IL) was used for data analysis. The receiver operating characteristic (ROC) curve determined the optimal cutoff value for EBV-DNA and SUV_max_ to predict outcome with the best trade-off between sensitivity and specificity. The distribution of the subgroups of locoregional recurrence and distant recurrence were skewed; hence, a Wilcoxon rank sum test of two independent samples was used to compare the value of EBV DNA and SUV_max_ between the two subgroups. Overall survival (OS) was calculated from the date of recurrence diagnosis to the date of death from any cause or patient censoring at the date of the last follow-up. Median OS values were estimated using the Kaplan-Meier method, and groups of interest were compared using log-rank tests. The univariate and multivariate survival analyses were based on the Cox proportional hazards regression model. Variables in the model included sex, age, family history of tumor, restage, number of metastases, EBV DNA, and SUV_max_. Spearman rank correlation was used to delineate the relationship between EBV-DNA, SUV_max_ and clinical variables. A P<0.05 from a two-tailed test indicated statistical significance.

## Results

### Baseline characteristics and survival time in the subgroups of patients with locoregional or distant recurrence

**Fig 1:**
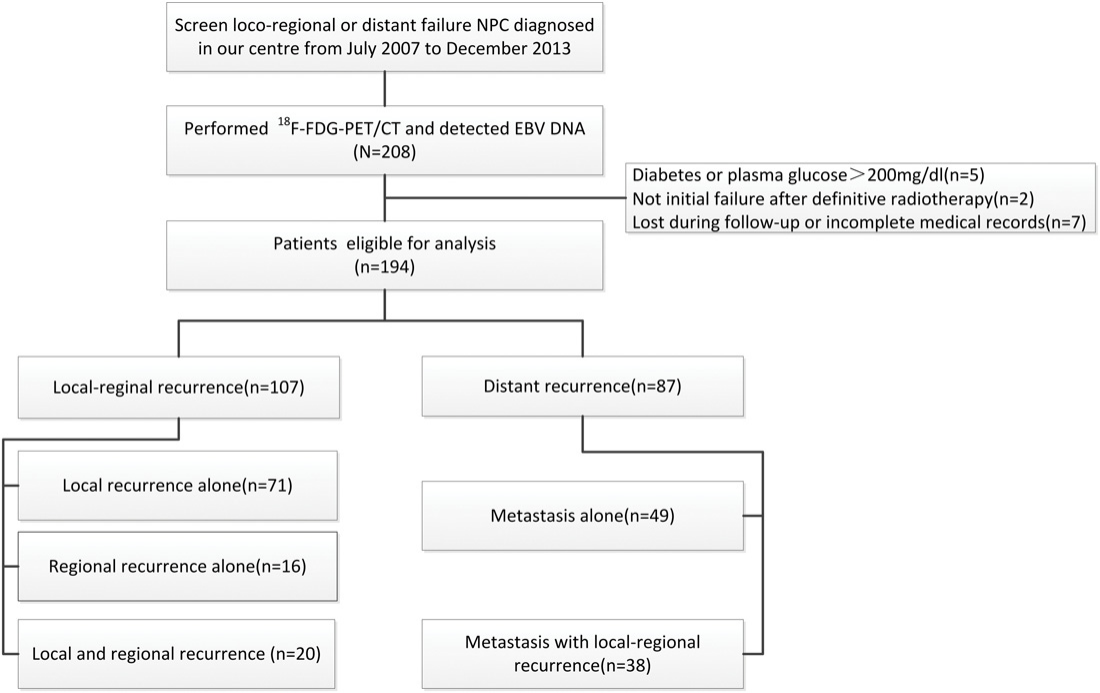
Flowchart of patients enrolled in this study.

A profile of this study is shown in Fig 1. Between July 2007 and December 2013, 208 patients were assessed and entered into this study. Fourteen cases were excluded: five due to diabetes or plasma glucose>200 mg/dl, two because there was not an initial failure after definitive radiotherapy, and the others due to attrition during follow-up or incomplete medical records. Therefore, 194 patients in all were eligible for analysis, among which 160 were men and 34 were women. Patient characteristics are described in Table 1. The primary histologic type of the 194 cases, 178 cases presented non-keratinizing undifferentiated carcinoma (formerly WHO type III), 4 cases presented non-keratinizing differentiated carcinoma (formerly WHO type II), only 1 presented keratinizing squamous cell carcinoma (type I) and 11 patients missed data. The mean age of all patients was 43.9 years (range, 10 to 70 years). Among the 194 patients, 107 had locoregional recurrence only, and 87 had distant failure with or without locoregional recurrence. 103 cases were diagnosed recurrence by pathology and 91 cases were diagnosed by imaging test. Based on whether the patients exhibited distant failure, all patients were divided into two subgroups, the locoregional recurrence group and the distant recurrence group. At the time of the final follow-up, 30 of 107 patients (28%) had died in the locoregional recurrence subgroup, whereas 31 of 87 patients (35.6%) had died in the distant recurrence subgroup. The median concentration of plasma EBV DNA was 4,000 copies/ml for patients with locoregional recurrence only, which was much lower than that of patients with distant metastasis (34,900 copies/ml, P<0.001). The median SUV_max_ for patients with distant recurrence was much higher than that of the locoregional recurrent patients (13.3 vs. 11.50, P<0.001). We found that EBV DNA could not be detected in 37 of 107 patients (34.6%) with locoregional recurrence only and 12 of 87 patients (13.8%) with distant recurrence. Spearman correlation analysis demonstrated that EBV DNA levels positively correlated with number of metastases and that SUV_max_ correlated with clinical restage and number of metastases (S1 Table).

**Table 1:** Characteristics of patients with recurrent or metastatic nasopharyngeal carcinoma after definitive radiotherapy.

Characteristic	Number of patients	
	Locoregional Recurrence (%)	Distant recurrence (%)
	(107)	(87)
Gender		
Female	21 (19.6)	13 (14.9)
Male	86 (80.4)	74 (85.1)
Age		
<46	58 (54.2)	56 (35.6)
≥46	49 (45.8)	31 (64.4)
Family history of tumor		
Negative	87 (79.8)	75 (86.2)
Positive	20 (20.2)	12 (13.8)
EBV DNA^※1^		
mean (copies/ml)	2.67×10^4^	2.58×10^6^
median (copies/ml)	4.00×10^3^	3.49×10^4^
Restage		
I	10 (9.3)	0
II	10 (9.3)	0
III	49 (45.9)	0
IV	38 (35.5)	87 (100)
SUV_max_ ^※2^		
mean	12.48	14.39
median	11.5	13.3
Histopathology		
WHO^※3^ 1	0	1
WHO 2	4	0
WHO 3	97	81

Abbreviations:

^※1^. EBV DNA, Epstein-Barr virus DNA;

_※2_. SUV_max_, the maximal standardized uptake value. For metastasis, SUV_max_ represents distant recurrent SUV_max_; for locoregional recurrence, SUV_max_ represents locoregional recurrent SUV_max_;

^※3^ WHO, world health organization.

**Fig 2:**
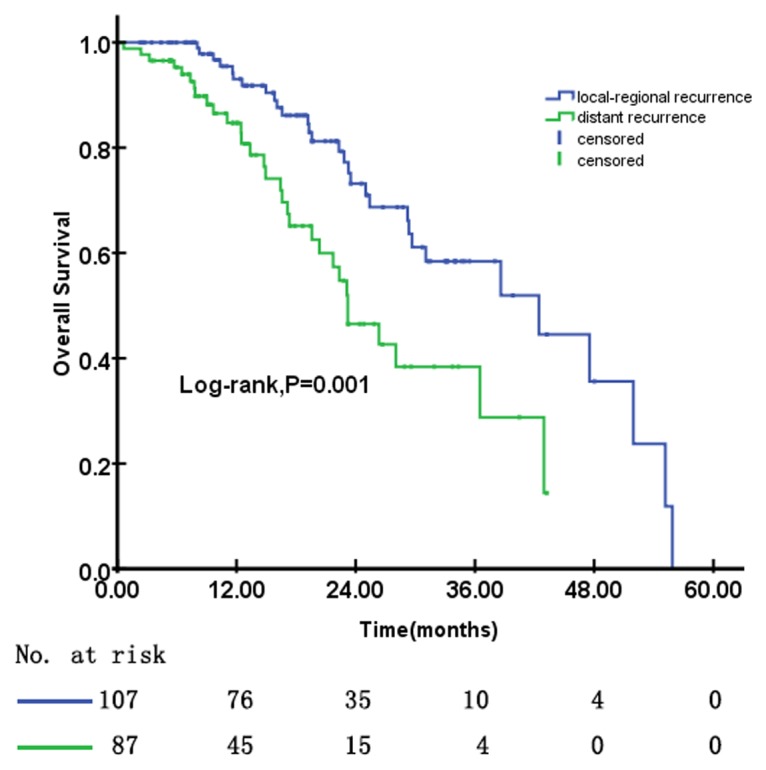
Kaplan–Meier estimates of overall survival between patients with locoregional recurrence and those with distant recurrence.

The mean follow-up time was 18.09 months (range, 0.62 to 55.88 months). Survival time was analyzed using the Kaplan-Meier method and compared with log-rank tests between the locoregional recurrence and distant recurrence subgroups. The results are shown in Fig 2. The median survival time for NPC patients with locoregional recurrence or distant recurrence was 42.45 months (95%CI, 26.11 to 58.79 months) and 23.20 months (95%CI, 18.49 to 27.90 months), respectively (P = 0.001, as shown in Fig 2).

### **Prognostic value of EBV DNA and SUV**_max_ **for patients with locoregional recurrence**

**Fig 3:**
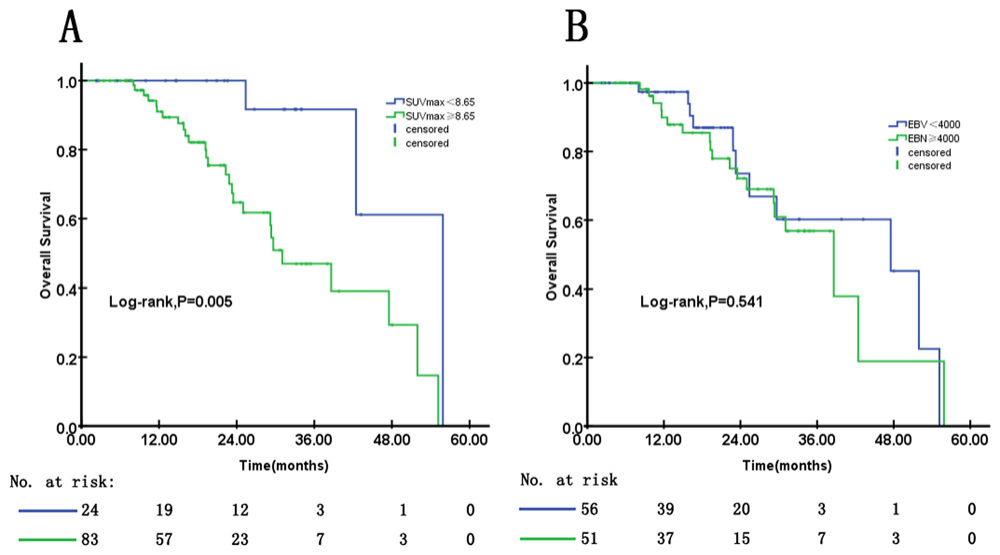
Kaplan–Meier estimates of overall survival according to the locoregional SUV_max_ or the plasma EBV DNA concentration in locoregional recurrent patients.

Next, we constructed ROC curves for death events to separately identify the impact of SUV_max_ and EBV DNA levels on the survival of locoregional recurrent and metastatic NPC patients. We used a series of cutoff points and finally selected the best cutoff values as the threshold for the subsequent binary variable analysis (Tables 2 and 3). With the value of 8.65 as the optimal cutoff point for patients with locoregional recurrence, patients with SUV_max_≥8.65 were found to have the most discriminate significance for OS (P = 0.005; Fig 3). The median OS was 31.05 months (95%CI, 22.76 to 39.33 months) for the patients with SUV_max_≥8.65, which was much shorter than that of the patients with SUV_max_< 8.65, who had a value of 55.88 months (95%CI, 39.77 to 58.70 months). Selecting the optimal cutoff point of 4000 copies/ml for EBV DNA, the Kaplan-Meier method demonstrated that there was no significant difference for OS between the low and high EBV DNA groups (P = 0.541; Fig 3). Univariate analysis showed that a locoregional SUV_max_≥8.65 was an unfavorable prognostic factor (P = 0.013; Table 2). To adjust for SUV_max_, the potentially associated parameters were introduced into the multivariable Cox regression model. The multivariate results confirmed that an SUV_max_≥8.65 was a highly significant predictor for OS for locoregional recurrent patients (HR = 4.882; 95%CI = 1.055–22.590; P = 0.042; Table 2), independent of restage, EBV DNA level, family history of tumor, gender and age. These results indicated that the predictive ability of SUV_max_ was superior to that of EBV DNA for predicting survival for patients with locoregional recurrence.

**Table 2:** Univariate and multivariate analyses of factors associated with survival in a cohort of 107 recurrent nasopharyngeal carcinoma patients after definitive radiotherapy.

Factors	Univariate	Multivariate	
	HR (95%CI) P value	HR (95%CI)	P value
Gender			
Female	Baseline		
Male	1.321(0.557–3.135) 0.528	0.779 (0.276–2.196)	0.636
Age			
<46	Baseline		
≥46	1.777 (0.844–3.740) 0.130	1.445 (0.617–3.387)	0.397
Family history of tumor			
Negative	Baseline		
Positive	1.060(0.399–2.814) 0.907	1.145 (0.392–3.343)	0.804
EBV DNA^※1^			
<4000 copiem/ml	Baseline		
≥4000 (copies/ml)	1.273(0.586–2.763) 0.542	1.793 (0.749–4.291)	0.19
Restage			
I-II	Baseline		
III-IV	6.642(0.893–49.416) 0.064	2.931 (0.354–24.283)	0.319
Locoregional SUV_max_ ^※2^			
<8.65	Baseline		
≥8.65	6.220 (1.470–26.31) 0.013	4.882 (1.055–22.590)	0.042

Abbreviations:

^※1^. EBV DNA, Epstein-Barr virus DNA;

^※2^. SUV_max_, the maximal standardized uptake value.

**Table 3:** Univariate and multivariate analyses of factors associated with survival in a cohort of 87 metastatic nasopharyngeal carcinoma patients with or without recurrence after definitive radiotherapy.

Factors	Univariate		Multivariate	
	HR (95%CI)	P value	HR (95%CI)	P value
Gender				
Female	Baseline			
Male	1.742 (0.602–5.043)	0.036	2.375 (0.678–8.315)	0.176
Age				
<46	Baseline			
≥46	2.117 (0.844–3.740)	0.046	2.687 (1.156–6.244)	0.022
Family history of tumor				
Negative	Baseline			
Positive	1.901 (0.771–4.685)	0.163	0.687 (0.262–1.802)	0.446
EBV DNA^※1^				
<21,100 (copies/ml)	Baselie			
≥21,100 (copies/ml)	2.608 (1.165–5.836)	0.020	3.269 (1.383–7.728)	0.007
Number of metastases				
<3 (n = 50)	Baseline			
≥3 (n = 37)	2.077 (1.021–4.224)	0.044	0.760 (0.297–1.944)	0.567
Distant SUV_max_ ^※2^				
<13.55	Baseline			
≥13.55	2.818 (1.302–6.098)	0.009	2.415 (0.956–6.101)	0.062

Abbreviations:

^※1^. EBV DNA, Epstein-Barr virus DNA;

^※2^. SUV_max_, the maximal standardized uptake value.

### **Prognostic value of EBV DNA levels and SUV**_max_ **for patients with distant recurrence**

**Fig 4:**
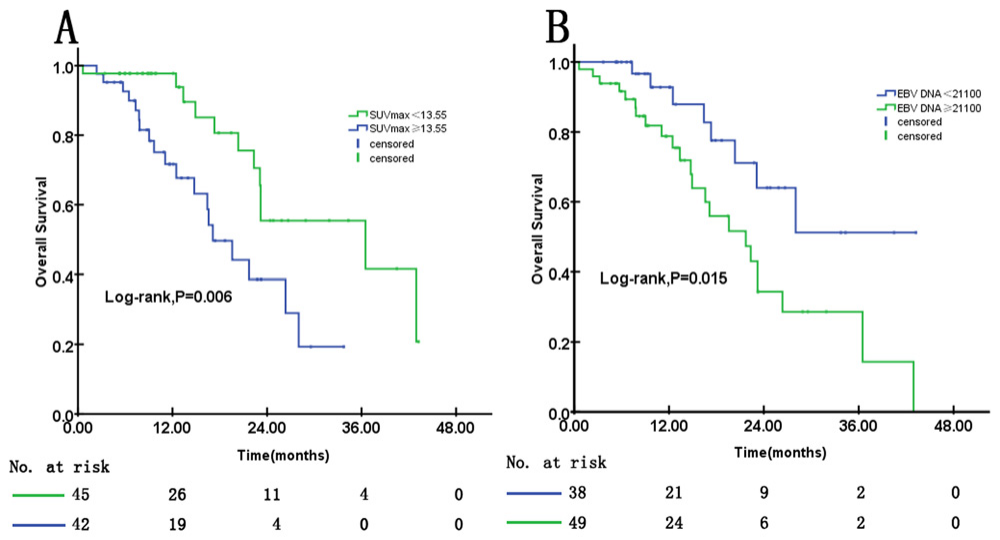
Kaplan–Meier estimates of overall survival according to the distant SUV_max_ or the plasma EBV DNA concentration in distant recurrent patients.

We found that ROC curves, which identified the impact of SUV_max_ and EBV DNA levels on the survival of metastatic NPC patients, had significantly different areas under the curve (AUCs). As S1 Fig shows, the AUC of EBV DNA was 0.664 (95%CI, 0.543 to 0.784), which is larger than that of SUV_max_, which had an AUC of 0.595 (95%CI, 0.455 to 0.735). The best cutoff was selected for metastatic NPC, and patients were categorized into two groups for separate Kaplan-Meier analysis according to their cutoff values of EBV DNA and SUV_max_. Fig 4 shows the Kaplan-Meier estimates for OS based on EBV DNA and SUV_max_. SUV_max_≥13.55 and EBV DNA≥21,100 copies/ml were two factors that significantly associated with shorter OS (P = 0.006 and P = 0.015, respectively; Fig 4). For the patients with SUV_max_≥13.55, the median OS was much shorter compared with the patients with SUV_max_<13.55, with values of 17.12 months (95%CI, 12.95–21.29 months) vs. 36.50 months (95%CI, 9.21–63.79 months), respectively (P = 0.006). Similarly, patients with EBV DNA≥21,100 copies/ml had a shorter median OS of 21.72 months (95%CI, 14.15–29.29 months) compared with 31.65 months (95%CI, 25.41–37.89 months, P = 0.015) in the group with EBV DNA<21,100 copies/ml. Univariate Cox proportional hazards regression analyses revealed that being male and having an age≥46, EBV DNA≥21,100 copies/ml, number of metastases≥3 and an SUV_max_≥13.55 was strongly associated with an unfavorable OS (Table 3). Multivariate Cox proportional hazards regression analyses further confirmed that age (HR = 2.687; 95%CI = 1.156–6.244; P = 0.022) and EBV DNA (HR = 3.269; 95%CI = 1.383–7.728; P = 0.007) were highly significant prognostic factors for OS, and SUV_max_ of patients with distant recurrence was borderline significant (HR = 2.415; 95%CI = 0.956–6.101; P = 0.062). These results showed that the predictive ability of EBV DNA was superior to that of SUV_max_ for predicting survival for patients with distant metastasis.

### **Prognostic value of SUV**_max_ **in patients with undetectable EBV DNA**

A total of 49 patients (37 of 107 with locoregional recurrences and 12 of 87 with distant recurrences) had undetectable EBV DNA levels. To assess the prognostic value of SUV_max_ in patients with undetectable EBV DNA levels, this special population was further analyzed using the Kaplan-Meier method and compared with log-rank tests. Because the analysis was limited by a small number of samples, patients with locoregional or distant recurrence with undetectable EBV DNA levels were analyzed together. A cutoff SUV_max_ of 8.65 was used for the analysis. The results are presented in S2 Fig. A higher SUV_max_ (SUVmax≥8.65) was strongly correlated with poor OS in recurrent patients with undetectable EBV DNA (P = 0.011). Multivariate analysis demonstrated that a level of SUV_max_≥8.65 was still a significant predictor for OS for recurrent patients with undetectable EBV DNA (HR = 5.093; 95%CI = 1.093–23.721; P = 0.038; S2 Table). These results illustrated that SUV_max_ is still an effective prognostic indicator for those with undetectable levels of EBV DNA.

## Discussion

Prognostic factors, which can be assayed noninvasively, are extraordinarily vital for cancer treatment stratification and potentially improving treatment outcome. The recent demonstration of the use of ^18^F-FDG-PET SUV_max_ and EBV DNA levels in NPC patients has opened up new possibilities for detecting and monitoring recurrent NPC patients in southern China and Southeast Asia.[9, 21] A study by Chang suggested that tumor TLG, nodal TLG, total TLG, tumor SUV_max_, and nodal SUV_max_ were all significantly associated with plasma EBV DNA load.[27] However, this correlation of SUV_max_ and EBV DNA was not found in this study of patients with recurrent NPC. We explored and compared the prognostic implication of SUV_max_ and EBV DNA in subgroups of locoregional recurrent or distant recurrent NPC patients in the present study. Intriguingly, we found that the value of SUV_max_ was an effective prognostic biomarker for overall survival in patients with locoregional recurrence and patients with undetectable EBV DNA levels. According to our findings, the predictive power of EBV DNA load was superior to that of SUV_max_, although both distant SUV_max_ and EBV DNA load were independent prognostic factors that predicted survival for patients with distant recurrence.

Studies are lacking on prognostic markers for patients with locoregional recurrence. Chan explored the value of SUV_max_ and EBV DNA in predicting the outcome of nasopharyngectomy and cervical lymphadenectomy for patients with locoregional recurrent NPC.[17] However, the study was limited as it was unable to predict prognosis, although it found that preoperative EBV DNA and SUV_max_ levels of tumors had a predictive value for surgical resection margin status. In this study of a subgroup of 107 locoregional recurrent NPC patients, we noted that a higher SUV_max_, rather than EBV DNA level, had significant clinical value for predicting poor OS. To our knowledge, previous studies indicated that EBV DNA was associated with the TNM stage and reflected the tumor burden. Although there are controversial opinions regarding from where the plasma/serum circulating EBV DNA is derived, an increasing number of studies indicate that EBV DNA originates from tumor lesions and correlates with tumor load.[28–30] In contrast to other head and neck cancer and epithelial malignancy in general, a unique feature of NPC is its strong association with EBV. The life cycle of EBV includes latent and lytic stages. In NPC tumor cells, EBV hides in a latent status and latent viral genomes are periodically reactivated to the lytic cycle.[31, 32] For the patients with advanced stage disease, more NPC cells were reactivated into lytic stage, and released quantities of EBV DNA fragment into the circulation by lysis death. [33]Patients with locoregional recurrence showed a relatively low tumor load and therefore displayed low EBV DNA levels, partially explaining why EBV DNA was not an effective prognostic indicator for locoregional recurrence. In contrast, SUV_max_ of a lesion tends to show its metabolic activity and thus the aggressiveness of the tumor.[34, 35] In addition, a systematic review of 1813 patients from the published literature showed that ^18^F-FDG PET/CT imaging was significantly superior to MRI and CT for the diagnosis of recurrent locoregional disease,[36] and according to our findings, the SUV_max_ of ^18^F-FDG PET/CT also serves as a valuable predictive factor. Furthermore, the overexpression of glucose transporter 1 (GLUT1) has been shown to be closely related to ^18^F-FDG uptake in human cancer,[37] and it has been widely reported that GLUT1 correlates with poor prognosis and tumor aggressiveness in lung[38] and colorectal[39] carcinomas and in squamous cell carcinoma of the head and neck[40]. Together, these results suggest a possible explanation for why the predictive ability of SUV_max_ was superior to that of EBV DNA levels in patients with locoregional recurrence. However, there were still some NPC cases accompanied concurrently by locoregional recurrence and subclinical distant metastasis. In this circumstance, the plasma EBV DNA test should be routinely performed to monitor disease progression during the retreatment and follow up.

Several studies have also been published concerning the predictive value of SUV_max_ and EBV DNA load for distant recurrence after treatment. These studies demonstrated that EBV DNA levels in metastatic/recurrent NPC could predict tumor response and patient survival.[15, 18]^,^ However, the two studies failed to reveal the definite pattern of recurrence (i.e., locoregional recurrence or distant recurrence). Chan revealed that SUV_max_ was an effective biomarker for predicting prognosis in metastatic NPC.[41] Consistent with these results, we found that patients with higher distant SUV_max_ values presented a worse overall survival compared with patients with lower distant SUV_max_ values. It has been reported that EBV DNA load is a better prognosticator for distant metastatic recurrence than locoregional recurrence in NPC.[42] Consistent with this previous report, we found that EBV DNA load is an independent prognostic factor for overall survival in patients with distant recurrence but not in patients with locoregional recurrence. It is also logical to suggest that the higher tumor burden in patients with distant recurrence accounts for the better predictive ability of the level of EBV DNA. Another alternative explanation for the better predictive ability of EBV DNA load in patients with distant recurrence compared with patients with locoregional recurrence is that distant recurrences originate from subclinical micro-metastases in the circulation that are seeded from the primary tumor before the commencement of radiation therapy.[42] We also found that the predictive ability of SUV_max_ is inferior to that of the level of EBV DNA for patients with distant recurrence. It is worth noting that SUV_max_ is only the reflection of metabolism activity of a locoregional site and cannot sufficiently reflect tumor cells in the circulation, which are known as micro-metastases. Thus, it is reasonable to hypothesize that for those with distant metastasis, there may be numerous micro-metastases in the circulation. Present imaging modalities are not sufficient to discover this micro-metastasis. However, the higher EBV DNA concentration in patients with distant recurrent NPC, as reflected in our results, might closely correlate with the degree of micro-metastasis.

In our analysis of 194 recurrent patients, 49 presented with undetectable EBV DNA levels, and further study showed that SUV_max_ was also predictive of survival for these patients. NPC patients with undetectable EBV DNA levels have been reported in previous studies. Yip et al. summarized previous studies and found that an EBV DNA qPCR achieved sensitivities of tumor detection of 22–86%, 48–95%, 74–100% and 79–100% in stage I, II, III and IV patients, respectively.[43] They further reviewed 6 studies from 2003 to 2013 that compared the ability of post-treatment EBV DNA loads to detect distant metastasis (DM) and local recurrence (LR) or locoregional recurrence (LRR). The detection rate for DM ranged from 55% to 96% and varied from 0% to 67% for LR/LRR. It is noteworthy that a low or undetectable EBV DNA load does not necessarily predict good prognosis. Nevertheless, SUV_max_ is an independent determinant to EBV DNA load in predicting survival for patients with recurrent NPC and undetectable EBV DNA.

However, some limitations remain in the present study. First, our study was limited by the relatively small sample size and short follow-up time. Second, the measurements were recorded from a single center. Third, the clinical application of quantitative plasma EBV DNA results has been limited by varying sensitivities when different segments of viral gene or different viral genes are assayed in different study group[44–46]. Hence, a larger, multicenter cohort study with unified standardized procedures is warranted.

In conclusion, we clearly demonstrated that pretreatment locoregional SUV_max_ level was the only independent predictive factor of OS for patients with locoregional recurrence, but for patients with distant recurrence, the predictive ability of EBV DNA load was superior to that of SUV_max_. In addition, SUVmax remains an effective indicator for patients with undetectable amounts of EBV DNA. This study may guide individualized treatment for recurrent NPC patients. Further prospective, multicenter investigations to evaluate the feasibility and role of SUV_max_ and EBV DNA concentration for prognostication in recurrent NPC are warranted.

## Supporting Information

S1 Fig Receiver operating characteristic (ROC) curves for EBV DNA and SUV_max_. (TIF)

S2 Fig Kaplan–Meier estimates of overall survival in 49 recurrent patients with undetectable EBV DNA levels. (TIF)

S1 Table Relationship between EBV DNA and SUV_max_
(PDF)

S2 Table Multivariate analyses of factors associated with survival in a cohort of 49 recurrent nasopharyngeal carcinoma patients with undetectable EBV DNA loads. (PDF)

S1 Dataset The origin dataset of the manuscript. (SAV)
