# Supplementary figures and images for: Different Prognostic Values of Plasma Epstein-Barr Virus DNA and Maximal Standardized Uptake Value of 18F-FDG PET/CT for Nasopharyngeal Carcinoma Patients with Recurrence
Source: PLoS One. 2015 Apr 8;10(4):e0122756. doi: 10.1371/journal.pone.0122756 (PMC4390333; doi:10.1371/journal.pone.0122756)

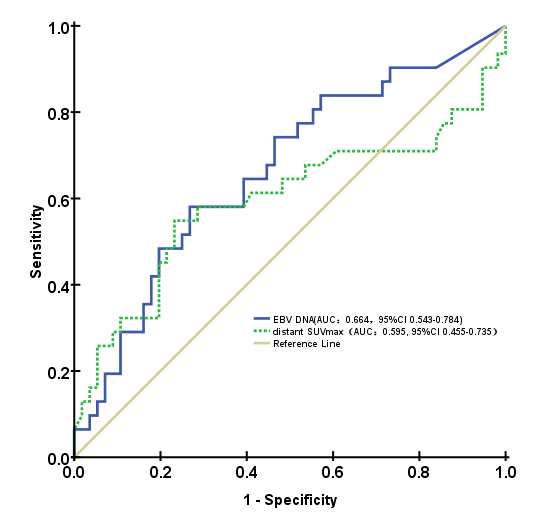

Supplement: S1 Fig — (TIF) [file pone.0122756.s001.tif]

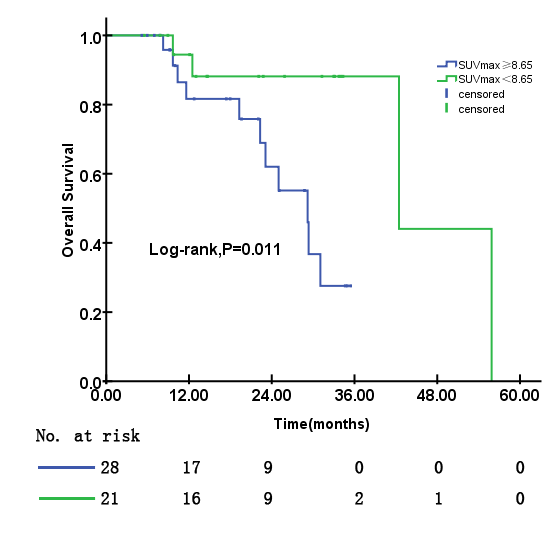

Supplement: S2 Fig — (TIF) [file pone.0122756.s002.tif]
