# Supplementary material for: Different Prognostic Values of Plasma Epstein-Barr Virus DNA and Maximal Standardized Uptake Value of 18F-FDG PET/CT for Nasopharyngeal Carcinoma Patients with Recurrence
Source: PLoS One. 2015 Apr 8;10(4):e0122756. doi: 10.1371/journal.pone.0122756 (PMC4390333; doi:10.1371/journal.pone.0122756)
Supplement: S1 Table — (PDF) [file pone.0122756.s003.pdf]

S1 Table. Relationship between EBV DNA and SUV_{max}

Variable	EBV DNA※1		SUVmax※2	
	Spearman correlation coefficient	P value	Spearman correlation coefficient	P value
**Locoregional recurrence**				
Gender (male/female)	-0.159	0.102	-0.091	0.353
Age (years)	0.086	0.379	-0.015	0.878
Family history of tumor (yes/no)	0.132	0.175	0.199	0.039
Restage (Ⅰ/ Ⅱ/ Ⅲ / Ⅳ)	0.081	0.408	0.325	0.001
EBV DNA (copies/ml)			0.113	0.246
Locoregional SUVmax	0.113	0.246		
**Distant recurrence**				
Gender (male/female)	-0.092	0.397	0	1.000
Age (years)	0.048	0.656	0.109	0.316
Family history of tumor (yes/no)	-0.025	0.816	-0.092	0.395
Number of metastases	0.415	0.000	0.263	0.014
EBV DNA (copies/ml)			0.143	0.185
Distant SUVmax	0.143	0.185		

Abbreviations: 1. EBV DNA, Epstein-Barr virus DNA; 2. SUVmax, the maximal standardized uptake value.
