# Supplementary material for: Different Prognostic Values of Plasma Epstein-Barr Virus DNA and Maximal Standardized Uptake Value of 18F-FDG PET/CT for Nasopharyngeal Carcinoma Patients with Recurrence
Source: PLoS One. 2015 Apr 8;10(4):e0122756. doi: 10.1371/journal.pone.0122756 (PMC4390333; doi:10.1371/journal.pone.0122756)
Supplement: S2 Table — (PDF) [file pone.0122756.s004.pdf]

S2 Table. Multivariate analyses of factors associated with survival in a cohort of 49 recurrent nasopharyngeal carcinoma patients with undetectable EBV DNA loads.

Factors	Overall survival	
	**HR (95% CI)**	**P value**
Gender: male vs female	1.021 (0.236-4.413)	0.978
Age: ≥46 vs <46	1.173 (0.336-4.097)	0.803
Restage: Ⅰ-Ⅱ vs Ⅲ-Ⅳ	2.065 (0.229-18.636)	0.518
Family history of tumor: yes vs no	0.944 (0.227-3.933)	0.937
SUVmax[※]: ≥8.65 vs <8.65	5.093 (1.093-23.721)	0.038

Abbreviation: SUVmax, the maximal standardized uptake value.
